# Motion Detection in Diffusion MRI via Online ODF Estimation

**DOI:** 10.1155/2013/849363

**Published:** 2013-02-21

**Authors:** Emmanuel Caruyer, Iman Aganj, Christophe Lenglet, Guillermo Sapiro, Rachid Deriche

**Affiliations:** ^1^Athena Project-Team, INRIA, 2004 Route des Lucioles, BP93, 06902 Sophia Antipolis, INRIA Sophia Antipolis Méditerranée, France; ^2^Martinos Center for Biomedical Imaging, Massachusetts General Hospital, Department of Radiology, Harvard Medical School, 149 13th Street, Charlestown, MA 02129, USA; ^3^LIDS, Department of Electrical Engineering and Computer Science, Massachusetts Institute of Technology, 77 Massachusetts Avenue, Room 32-D608, Cambridge, MA 02139, USA; ^4^Center for Magnetic Resonance Research, Department of Radiology, University of Minnesota Medical School, 2021 Sixth Street SE, Minneapolis, MN 55455, USA; ^5^Department of Electrical and Computer Engineering, University of Minnesota, 200 Union Street SE, Minneapolis, MN 55455, USA

## Abstract

The acquisition of high angular resolution diffusion MRI is particularly long and subject motion can become an issue. The orientation distribution function (ODF) can be reconstructed online incrementally from diffusion-weighted MRI with a Kalman filtering framework. This online reconstruction provides real-time feedback throughout the acquisition process. In this article, the Kalman filter is first adapted to the reconstruction of the ODF in constant solid angle. Then, a method called STAR (STatistical Analysis of Residuals) is presented and applied to the online detection of motion in high angular resolution diffusion images. Compared to existing techniques, this method is image based and is built on top of a Kalman filter. Therefore, it introduces no additional scan time and does not require additional hardware. The performance of STAR is tested on simulated and real data and compared to the classical generalized likelihood ratio test. Successful detection of small motion is reported (rotation under 2°) with no delay and robustness to noise.

## 1. Introduction

Diffusion MRI has provided a great tool for neuroscientists to understand and analyze *in vivo* the anatomy of the brain white matter fiber tracts that connect different areas of the cortex. The diffusion tensor model [1] has become increasingly popular, and the study of scalar indices derived from it has proved useful in the diagnosis of a wide range of neurological diseases [2, 3]. For several specific applications, like fiber tractography, this model is, however, known to be limited, and high angular resolution imaging techniques should be used instead, to reconstruct the model-free ensemble average propagator [4–6] or the orientation distribution function (ODF) [7–10].

The acquisition of high angular resolution diffusion images requires longer time than diffusion tensor imaging. Subjects are likely to move during these acquisitions, and we can identify at least three motivations to develop a proper method for the online detection of motion. First, images can be registered prior to diffusion model estimation; however this might increase partial volume effects [11], because of the relatively low spatial resolution of diffusion-weighted images and of interpolation in the registration procedure. This also modifies the variance properties of the image [12], which should be considered carefully for group studies. When the subject moves during acquisition, a warning could be issued, so as to take a decision accordingly. Depending on the number of images already acquired, the decision could be to restart the scan or acquire a few more diffusion weighted images than originally planned to compensate for the variance increase due to the registration. Second, diffusion acquisitions use a gradient table, which is a set of orientations and *b*-values and has been designed following an optimal sampling strategy. In Q-ball imaging for instance, the set of orientations is designed to sample the sphere in an optimal isotropic fashion [13, 14]. When correcting for motion, the diffusion encoding gradients should be rotated to be consistently defined in a coordinate frame related to the subject [15–17]. This modification might break the optimal sampling strategy as originally planned and affect the reconstruction of the ODF. Finally, in the context of online processing of diffusion images, motion must be detected, so that it can be corrected to continue the incremental reconstruction.

 Several solutions for online motion detection and correction were recently proposed [18–21]. The authors in [18] use a camera inside the scanner to detect and evaluate a rigid motion. Their study shows the improvement in ODF reconstruction with this prospective approach for motion correction over a classical offline registration. However, this technique requires additional hardware which is to date not always available on scanners. Other approaches [19, 21] are based on the interleaving of echo navigators through the acquisition sequence. The authors in [19, 21] report good results in detecting and correcting motion, but these additional acquisitions affect the overall protocol time. Finally, a recent work [20] introduces a motion detection and prospective motion correction to account for slow motion artifacts such as image misalignment. They also reduce fast motion effects such as signal dropout, by identifying the volumes most affected by motion, and schedule reacquisition at the end of the scan. This last technique is very promising and shows good results. But the motion detection and correction is performed by comparing a diffusion weighted image to the average of diffusion weighted images with the same *b*-value. We believe this is suboptimal, as it does not take into account the direction associated to each diffusion weighted image. This therefore might lead to a loss of sensitivity and specificity.

In this work, we propose a diffusion weighted-image-based technique for the online detection of motion in Q-ball imaging. Our method does not require new hardware or change in the acquisition protocol and is based on a Kalman filter reconstruction of the HARDI signal [22, 23]. The first contribution is the adaptation of the Kalman filtering framework for online reconstruction of ODF in constant solid angle recently introduced in the Q-ball imaging community [9, 10]. Then, we present a solution to the detection of motion in diffusion images, adapted from the generalized likelihood ratio test (GLRT) [24]. To overcome certain shortcomings of this method, we introduce STAR (STatistical Analysis of Residuals), an original method for the detection of motion in diffusion weighted images. The method is tested under various experimental conditions on semiartificial and on real data and compared to GLRT. In the Results, we report successful detection of small motion (rotation by angle under 2°), even in noisy conditions. The detection using STAR outperforms GLRT, while STAR does not need any delay for the detection.

## 2. Methods

In this section, we review the definition and the expression of the ODF calculated in constant solid angle. It has been shown recently that this mathematically correct definition of ODF can be reconstructed in Q-ball imaging [9, 10]. We present an incremental reconstruction algorithm for this ODF, based on the Kalman filter. We formalize the problem of motion detection only from the observation of the diffusion signal. We present a brief review of methods for fault detection, in particular GLRT, built upon the Kalman filter, as first described by [24]. Finally, we present STAR, an original approach based on a statistical modeling of the image. It has several advantages over GLRT. Both algorithms are implemented, and we present at the end of this section the validation methods used to compare them.

### 2.1. ODF in Constant Solid Angle

The ODF is a spherical function, retaining the angular information of the ensemble average propagator, *P*. When defined as the marginal probability of direction, the ODF, *ψ*, is the probability for a water molecule to diffuse along a given direction in a constant solid angle. It is defined from the diffusion propagator as

(1) $$\begin{array}{c} \psi \left(u\right)=\int_{0}^{\infty }P\left(ru\right)r^{2}\text{d}r. \end{array}$$

In diffusion MRI, we measure the signal, *s*(**q**), which is related to the ensemble average propagator *P* through a Fourier transform, under the narrow-pulse condition [25]

(2) $$\begin{array}{c} P\left(r\right)=\int_{q\in {\mathbb{R}}^{3}}\frac{s\left(q\right)}{s\left(0\right)}e^{-2\pi iq\cdot r}\text{d}q. \end{array}$$

Under the assumption of a monoexponential decay of the diffusion signal *s*, the relation between *s*(**q**), *s*(0), and the ODF *ψ* is given by

(3) $$\begin{array}{c} \psi \left(u\right)=\frac{1}{4\pi }+\frac{1}{16\pi^{2}}\text{FRT}\left\{\nabla_{b}^{2}\ln\left(-\ln\frac{s}{s\left(0\right)}\right)\right\}\left(u\right), \end{array}$$

where FRT denotes the Funk-Radon Transform and ∇_*b*_^2^ the Laplace-Beltrami operator [9].

The computation of the ODF can be implemented using the modified spherical harmonic basis for real and symmetric functions [8] to describe the transformed signal *y* = ln⁡(−ln⁡(*s*/*s*(0))) [9], as both the Funk-Radon transform and the Laplace-Beltrami operations in (3) have a close-form matrix expression in the spherical harmonic basis. If ${\hat{c}}_{j}$ are the spherical harmonic coefficients that describe *y*, then the spherical harmonic coefficients to describe the ODF *ψ* are

(4) $$\begin{array}{c} {\hat{c}}_{j}^{\prime }=\{\begin{array}{cc} \frac{1}{2\sqrt{\pi }} & j=1\\ -\frac{1}{8\pi }{\left(-1\right)}^{l_{j}/2}\frac{1\times 3\times \cdots \times \left(l_{j}+1\right)}{2\times 4\times \cdots \times \left(l_{j}-2\right)}{\hat{c}}_{j} & j>1, \end{array} \end{array}$$

where *l*_*j*_ = {0,2, 2,2, 2,2, 4,4, 4,…} for *j* = {1,2, 3,…} is the order associated to the *j*th spherical harmonic.

The computation of the spherical harmonic coefficients $\hat{c}$ describing *y* from a series of measurements *y*[*k*] = ln⁡(−ln⁡(*s*[*k*]/*s*(0))) = ln⁡(−ln⁡(*s*(*q* **u**[*k*])/*s*(0))), *k* = 1 ⋯ *N* at discrete positions **u**[*k*] on the unit sphere, and a measurement without any diffusion encoding gradient *s*(0) is implemented by minimizing:

(5) $$\begin{array}{c} M\left(c\right)={\left(y-Bc\right)}^{\text{T}}\Sigma^{-1}\left(y-Bc\right)+\lambda c^{\text{T}}Lc. \end{array}$$

**Figure 1:**
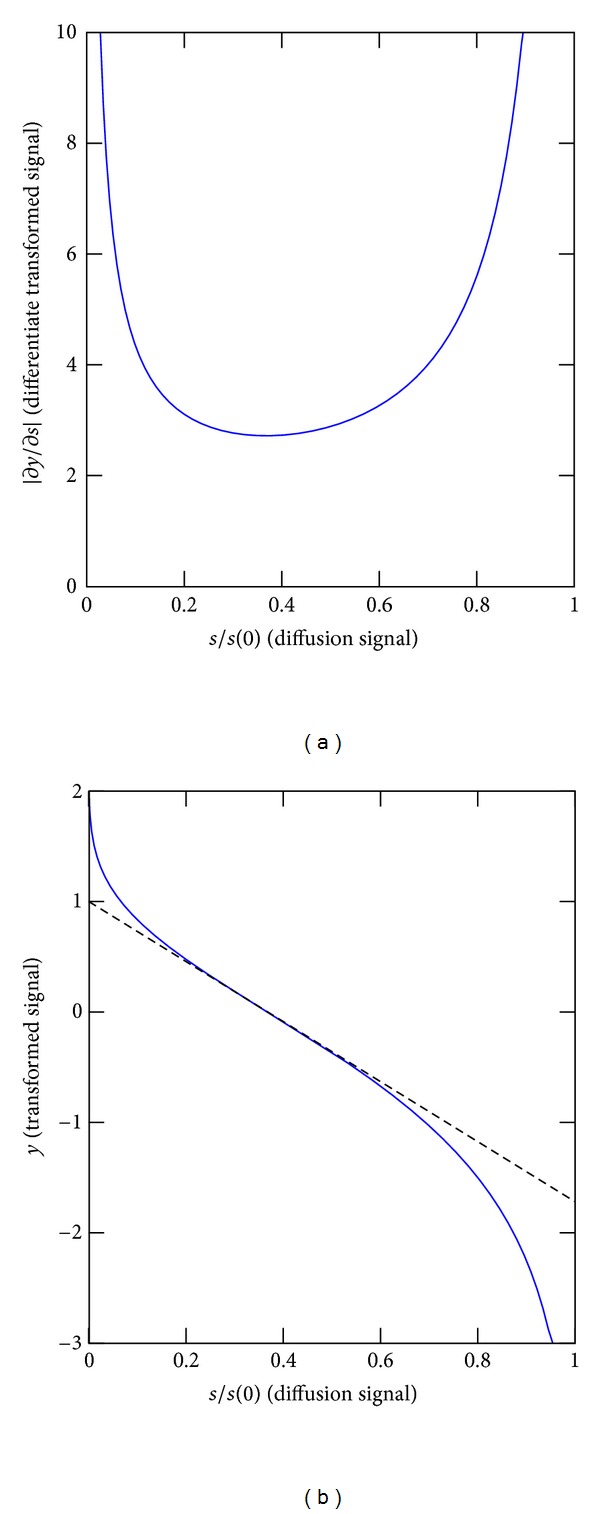
(a) Nonlinear transform on the diffusion signal; (b) derivative with respect to the signal. The distortion is maximum for *s*/*s*(0) → 0.0 and *s*/*s*(0) → 1.0 and minimum for *s*/*s*(0) ≈ 1/*e*.

The second term is a Laplace-Beltrami regularization constraint on the fitted signal, with the matrix **L** having diagonal elements *l*_*j*_^2^(*l*_*j*_+1)^2^. The matrix Σ in the data fitting term of (5) accounts for the uncertainty in the diffusion-weighted measurements *s*[*k*] as well as for the distortion introduced by the nonlinear transform, which is illustrated in Figure 1. The distortion is higher in high-magnitude and low-magnitude signal modes. The diagonal elements of Σ can be approximated through first-order error propagation; the uncertainty on the transformed signal *y* is simply

(6) $$\begin{array}{c} \delta y=\frac{\partial y}{\partial s}\delta s=-\frac{1}{s\ln\left(s/s_{0}\right)}\delta s. \end{array}$$

Provided that the error on separate measurements is uncorrelated, the diagonal elements *σ* ^2^[*k*] of Σ are simply

(7) $$\begin{array}{c} \sigma^{2}\left[k\right]=\frac{\mathrm{Var}\left(s\left[k\right]\right)}{{s\left[k\right]}^{2}\ln^{2}\left(s\left[k\right]/s_{0}\right)}, \end{array}$$

where Var⁡(*s*[*k*]) denotes the variance of the diffusion signal *s*[*k*] and can be estimated once for the whole volume using a method like PIESNO for instance [26].

### 2.2. Incremental ODF Reconstruction

Provided that the acquisition sequence is incremental (in this study we use the incremental point sets as in [23]), the energy in (5) can be minimized incrementally using a Kalman filter [23]. The incremental system adapted to the reconstruction of the ODF in constant solid angle is given by

(8) Initialization{c[0]=𝔼[c]P~[0]=𝔼[(c−c[0])(c−c[0])T]P[0]=(P~[0]−1+λL)−1,Update{V[k]=B[k]P[k−1]B[k]T+σ2[k]g[k]=P[k−1]B[k]TV[k]−1P[k]=(I−g[k]B[k])P[k−1]γ[k]=y[k]−B[k]c[k−1]c[k]=c[k−1]+g[k]γ[k].

**Figure 2:**
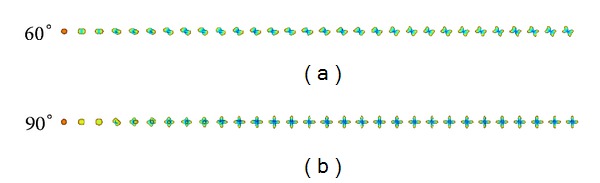
Synthetic mixture of Gaussian model, and reconstruction of the ODF in constant solid angle using the Kalman filter. (a) 60° and (b) 90° crossing fibers. The 30 first iterations of the Kalman filter are shown.

The *σ* ^2^[*k*] depend on the data as expressed in (7), and the covariance *V*[*k*] of the residual *γ*(*k*) can no longer be precomputed offline. The expected covariance of the estimated spherical harmonic coefficients **c**[*k*] is the matrix **P**[*k*] computed by the Kalman filter. Then the expected mean squared error on the spherical harmonic coefficients describing the ODF is given by

(9) $$\begin{array}{c} \text{MSE}\left(c^{\prime }\left[k\right]\right)=\mathrm{Tr}\left(F^{\text{T}}L^{\text{T}}P\left[k\right]LF\right), \end{array}$$

where **F** is the matrix form of the Funk-Radon transform and has diagonal elements 2*πP*_*l*_*j*__(0), where *P*_*l*_*j*__(0) is the Legendre polynomial of degree *l*_*j*_ evaluated at 0, and **L** is the Laplace-Beltrami matrix as in (5). An example of ODF reconstructed incrementally is shown on Figure 2.

The Kalman filter was derived with the assumption that the local diffusion propagator does not change in time. Next, we show how we can derive a motion detection algorithm from this Kalman filter.

### 2.3. Motion and Diffusion Signal

Subject motion generally occurs in a short time compared to the acquisition time. This may induce an *abrupt* change in the diffusion signal. The detection of abrupt changes in dynamical systems has been extensively studied [24, 27]; a very good review of methods and algorithms can be found in [28]. They propose a classification of change detection problems, together with suggested methods and algorithms to address them.

In the previous section, we have introduced a Kalman filter solution to reconstruct the spherical harmonic coefficients of the Q-ball signal. The state of our system is the vector of spherical harmonic coefficients **c**[*k*], and a motion of the subject at time *θ* is likely to imply a modification of this state, **c**[*k* ≥ *θ*] = **c**[*k* < *θ*] + **p**. The problem of motion detection reduces to the problem of change detection in this multidimensional system. Besides, since both the time *θ* and the magnitude **p** of the change are unknown *a priori*, the classification in [28] suggests to use a generalized likelihood ratio test (GLRT) for the detection. In the next section we briefly describe this method and its implementation upon a Kalman filter, as originally introduced in [24].

#### 2.3.1. Classical Solution: The Generalized Likelihood Ratio Test

The Kalman filter presented in the first section is built under the hypothesis of no motion. We can monitor the residuals of this Kalman filter for each iteration and test whether the hypothesis is still valid. As it has been shown in [24], the prediction error after a change occurred at time *θ* for subsequent iterations can be decomposed as

(10) $$\begin{array}{c} \gamma \left[k\right]=G\left(k,\theta \right)p+\gamma_{1}\left[k\right], \end{array}$$

where *γ*_1_ is zero-mean Gaussian distributed with covariance *V*[*k*] and **G**(*k*, *θ*) represents the propagation of a jump at time *θ*, to the prediction error at time *k*. This can be computed as in [24]:

(11) G(k,θ)=B[k](I−∑j=θk−1g[j]G(j,θ)),G(k,k)=B[k].

The problem of a change detection is to discriminate between two hypotheses:

(*ℋ*_0_): no change in the state vector: *γ*[*j*] = *γ*_1_[*j*], *j* = *θ*_0_ ⋯ *k*,
(*ℋ*_1_): at time *θ*_0_, the state vector becomes **c** + **p**_0_.

When **p**_0_ and *θ*_0_ are known, a natural statistic for the detection is the likelihood ratio. Provided that the residuals are linearly related to the change (10), the log-likelihood ratio is

(12) $$\begin{array}{c} l\left(k;\theta_{0},p_{0}\right)=\ln\frac{p_{{ℋ}_{1}}\left(\gamma \left[\theta_{0}\cdots k\right]\right)}{p_{{ℋ}_{0}}\left(\gamma \left[\theta_{0}\cdots k\right]\right)}. \end{array}$$

Provided that the densities *p*_*ℋ*_0__ and *p*_*ℋ*_1__ are Gaussian, after simplification this is rewriten as

(13) $$\begin{array}{c} l\left(k;\theta_{0},p_{0}\right)=\sum_{j=\theta_{0}}^{k}\gamma \left[j\right]V^{-1}\left[j\right]G\left(j,\theta_{0}\right)p_{0}. \end{array}$$

In our case, both **p** and *θ* are unknown. The generalisation of the likelihood ratio method suggests to replace *θ*_0_ and **p**_0_ in (13) by their maximum likelihood estimates:

(14) θ^(k)=argmax⁡θ l(k;θ,p^(k;θ)),p^(k;θ)=(∑j=θkGT(j,θ)V[j]−1G(j,θ))−1 ×∑j=θkGT(j,θ)V[j]−1γ[j]  (least squares estimate).

Finally, the decision is taken by comparing $l(k;\hat{\theta },\hat{p})$ to a threshold *ϵ*.

This technique works fine, yet suffers from several drawbacks. First, the calculation of the maximum likelihood estimate of **p** involves the inversion of a matrix in (14) which has full rank only if *k* − *θ* > dim⁡(**p**). In other words, this implies a delay at least equal to the dimension of the problem. As an example, when the signal is fitted in the 4th order symmetric spherical harmonic basis, this dimension is 15. In addition, the choice of a threshold *ϵ* was reported to be critical and highly dependent on the delay [29]. Finally, in our situation the state vector represents the diffusion signal locally, and GLRT does not say how to combine the statistics of different voxels to calculate a statistic which could be an indicator of motion for the whole volume at once. To address these weaknesses, we propose in the next section an original approach without delay, incorporating a statistical model of the image, in order to provide a more suitable detection algorithm.

#### 2.3.2. Statistical Analysis of the Residuals

The reconstructed image is a vector field **c**(**r**), where **c** is a vector of spherical harmonic coefficients describing the diffusion signal at voxel position **r**. We consider the difference **p** between two such vector fields **c**_1_ and **c**_2_, representing the same subject before and after a rigid transform. In what follows, we consider **p**(**r**) as a random variable, with unknown covariance matrix **C**.

Hence if there is no motion, the residuals for the whole volume will be distributed as *𝒩*(0, *V*[*k*]), where *V*[*k*] is the variance predicted by the Kalman filter. Otherwise the overall variance of the residuals will increase as *V*[*k*] + **G**(*k*, *θ*)**C** **G**(*k*,*θ*)^T^, where **G**(*k*, *θ*) is the matrix for the propagation of a jump at time *θ* to the prediction error at time *k*, and the covariance matrix **C** of **p** is unknown.

Based on the previous observations, we design a test for motion detection without delay. This means that based on measurements up to time *k*, we are able to decide whether a motion occurred at time *k* or not. Given a sample of *M* residuals at time *k*, at voxel positions **x**_1_ ⋯ **x**_*M*_ selected randomly within the brain, the hypotheses that a motion did occur at time *θ* or not are equivalent to

 (*ℋ*_0_) : *γ*[*k*] has variance *V*[*k*], as predicated by the Kalman filter;
 (*ℋ*_1_) : *γ*[*k*] has a variance *σ* ^2^ > *V*[*k*].

This decision problem is commonly addressed with a one-sided *χ* ^2^-test [30]. We first calculate the statistic:

(15) $$\begin{array}{c} T=\frac{\sum_{j=1}^{M}\gamma^{2}\left(x_{j}\right)\left[k\right]-{\left(1/M\sum_{j=1}^{M}\gamma [k]\right)}^{2}}{V\left[k\right]}. \end{array}$$

Under the hypothesis (*ℋ*_0_), *T* approximately follows a *χ*_*M*−1_^2^ distribution. We want to reject the hypothesis with a significance level *α*: under the hypothesis (*ℋ*_0_), we compute *p* such that *ℙ*(*T* > *p*) = *α*. The value of *p* is obtained from the inverse cumulative function of the *χ*_*M*−1_^2^ distribution.

### 2.4. Validation Methods

 We implemented the incremental reconstruction using Kalman filtering, together with GLRT and STAR for motion detection. These techniques were tested on real data, and a quantitative analysis of both was performed on semiartificial data, where the motion is simulated by a rigid transform. In this section, we describe how these images were synthesized.

The simulation is based on a tensor field reconstructed images of still subject, acquired on a 3T Siemens magnet at the Center for Magnetic Resonance Research, University of Minnesota Medical School, with 200 encoding directions computed following the optimal sampling scheme of [23], *b* = 1000 s/mm^2^, isotropic resolution 2.0 × 2.0 × 2.0 mm, 25 *b* = 0 images, 128 × 128 image matrix, 64 slices, TE = 90 ms, and TR = 8500 ms. We choose a series of diffusion gradient directions {**g**[*k*], *k* = 1 ⋯ *N*} and a *b*-value for synthesis. The rigid motion is specified by an instant *θ*, its rotation component **R** and its translation vector **t**. Provided that the diffusion encoding gradients should be rotated accordingly [15–17], the gradient directions used for synthesis are {**g**[1], **g**[2],…, **g**[*θ* − 1], **R** **g**[*θ*],…, **R** **g**[*N*]}. The rigid transform is finally applied to the synthetic diffusion weighted images *θ*,…, *N*, after which the images are corrupted by Rician noise.

## 3. Results

We evaluate the general likelihood ratio, and the residual-based statistics computed for STAR as a motion detection criterion. We first investigate the accuracy of the theoretical threshold in STAR. Then we compare the sensitivity and specificity of GLRT and STAR, for different values of the experimental parameter. Within this section, we report the true positive rate (TPR), defined as TPR = #detected positives/#positives, and the false positive rate, define as FPR = #mislabeled negatives/#negatives.

### 3.1. Software Implementation

 The Kalman filter and STAR were implemented in Python, with the use of the SciPy [31] toolkit, which is an efficient library for scientific computing. Based on this implementation, the reconstruction of the ODF field for an image of dimensions 128 × 128 × 64, with 200 diffusion-weighted images, took approximately 29 s on a 4-core Intel Core computer at 3.20 GHz, with 4.0 GB memory, running Linux Mint 13. This means that each diffusion volume is processed within less than 150 ms, which is short with respect to TR.

### 3.2. Threshold Selection in Motion Detection

 One of the advantages of STAR outlined in the previous section is that the threshold for the detection can be deduced from the target false positive rate. In practice, as *M* becomes large, we approximate the *χ*_*M*−1_^2^ distribution for the decision test described in Section 2.3.2 by a normal distribution: $\left(T-M+1)/\sqrt{2(M-1)}\sim 𝒩(0,1\right)$, and *p* is given by the inverse normal cumulative density function. For a false positive rate fewer than 5%, the theoretical threshold is $(p-M+1)/\sqrt{2(M-1)}=1.64$. This value is experimentally tested, and the results are presented in the next section.

**Figure 3:**
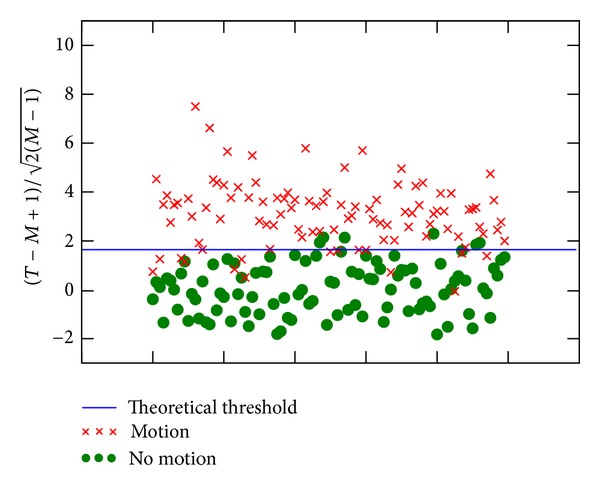
STAR statistics for simulations with and without motion. Each point represents a simulation; the theoretical threshold was calculated as explained in Section 2.3.2, so that the FPR does not exceed 5%.

We report in Figure 3 the value of the statistics $(T-M+1)/\sqrt{2(M-1)}$, for a series of 100 experiments without motion and a series of 100 experiments where the volume was rotated after 18 acquisitions by an angle of 2°. The threshold was taken as 1.64, for which the false positive probability is 5%. The empirical false positive rate we report for these 200 simulations is 4%, while the true positive rate is 90%.

### 3.3. Motion Detection: Sensitivity and Specificity

**Figure 4:**
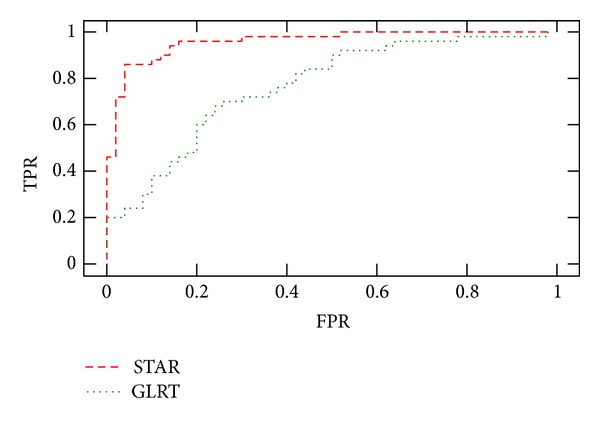
TPR versus FPR in motion detection. This experiment was done on 100 datasets without motion, and 100 acquisitions for which a rigid motion (rotation by an angle of 2°) occurred after 20 diffusion weighted images were acquired.

We computed both detection criteria on a series of 100 datasets without motion, and a series of 100 experiments with motion. We plot in Figure 4 the curve TPR *versus* FPR obtained by choosing different threshold values.

**Figure 5:**
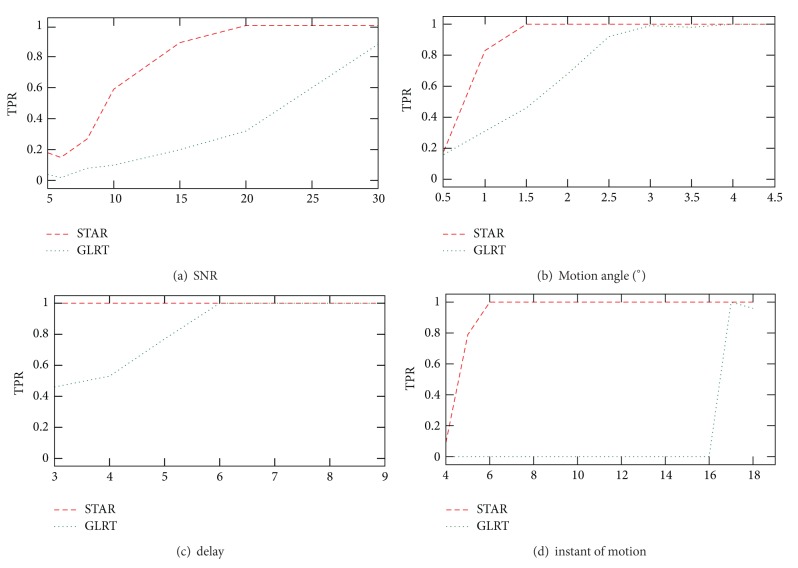
TPR: the threshold was chosen so that FPR = 5%. We compare the performance of GLRT and STAR. The dependency on several experimental conditions is tested: (a) SNR, (b) motion magnitude, (c) delay of the detection, and (d) instant of motion.

We also evaluate the robustness of GLRT and STAR in various experimental conditions. For a fixed FPR = 5%, we plot the TPR score of both methods. The experimental conditions include the delay, instant of motion in the acquisition sequence, SNR, and motion magnitude. The results of these simulations are reported in Figure 5. The experimental conditions, unless explicitly modified, were a rotation around the left-right axis by an angle of 3°, SNR = 20, motion instant *θ* = 20 and a delay *k* − *θ* = 3 (for GLRT). The experiment includes 400 negatives (simulations without motion) and 100 positives (simulations with motion). The monitoring of residuals in STAR and in GLRT is limited to 500 voxels randomly selected within the brain to get a computational cost adequate for online treatment.

#### 3.3.1. Experiment on a Real Data

**Figure 6:**
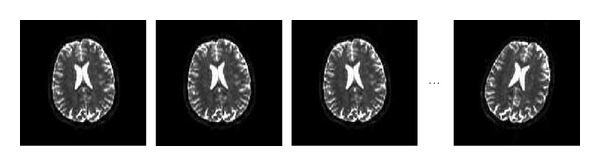
A real acquisition: the subject was asked to slightly move his head during the acquisition.

We also validate our methods on a real dataset, with the same imaging parameters as above. During the acquisition, the subject was asked to slightly tilt his head after 80 images were acquired. The motion was *a posteriori* identified as a rotation of 20° about the *z*-axis (see Figure 6). The detection algorithms could detect this motion: with a delay of 2 acquisitions for GLRT, and with no delay for STAR.

## 4. Discussion

Among the challenges of a motion detection algorithm, we have tested both GLRT and STAR in these conditions:

- small delay for the detection (ideally no delay);
- motion that occurred in the first few iterations of the Kalman filter;
- very noisy conditions (SNR down to 10);
- small motion.

With the help of simulated motion, we were able to present this exhaustive set of tests and subsequently the quantitative results. In addition to these tests on artificially added motion, we also showed the application of the method in a real setting, with a healthy subject asked to move on purpose during the scan.

Both criteria show good results in detecting motion, even in severe experimental conditions. As expected, STAR is more robust to noise (Figure 5(a)) and performs better in detecting small motion (Figure 5(b)), since it combines natively the residuals from different voxels.

In addition, GLRT cannot be computed if the number of acquired signals is lower than the dimension of the model, which is 15 in the case of 4^th^ order real, symmetric spherical harmonics. This impacts the ability to detect motion occurring at the beginning of an acquisition sequence: they are detected by STAR, while GLRT cannot be computed (see Figure 5(d)). In addition, GLRT needs a delay greater than 6 to get similar sensitivity as STAR (Figure 5(c)). STAR does not need any delay in the decision. Therefore, STAR is an original method that is best adapted to the problem of detecting motion online from a set of diffusion weighted images.

 As we have reported in the implementation section, the reconstruction of the ODF field using Kalman filtering is fast and compatible with online implementation.

## 5. Conclusions

In this paper, we have proposed a method for the detection of motion in diffusion MRI. We have developed a Kalman filter solution for the estimation of the ODF in constant solid angle. The detection algorithm STAR is based on the analysis of the residuals of the Kalman filter, yet it is general and can be directly applied to any linear diffusion model reconstruction. Compared to other techniques for the prospective detection and correction of motion [18, 19], our method does not require any camera or additional device. Once motion is detected by our technique, a decision could be taken by the scanner operator, or the protocol in [19] could be used for motion correction. To the best of our knowledge, our technique is the only image-based approach that clearly takes into account the directional information in diffusion weighted images to detect motion online.

The proposed technique was tested on semi-artificial data as well as in a real data and shows good results for the online detection of motion. Compared to GLRT, which is a classical solution for the detection of changes in dynamical systems, STAR combines the residuals at different voxel positions to compute a statistic, on which the decision is based. STAR performs better than GLRT in the detection of small motion, motion in noise, or motion occurring early in the acquisition protocol. Besides, STAR does not need any delay for the detection, which makes it very efficient in practical situations.
